# Development of the Dog Attachment Insecurity Screening Inventory (D-AISI): A Pilot Study on a Sample of Female Owners

**DOI:** 10.3390/ani11123381

**Published:** 2021-11-26

**Authors:** Giacomo Riggio, Marc Noom, Angelo Gazzano, Chiara Mariti

**Affiliations:** 1 Department of Veterinary Sciences, University of Pisa, 56124 Pisa, Italy; angelo.gazzano@unipi.it (A.G.); chiara.mariti@unipi.it (C.M.); 2 Research Institute of Child Development and Education, University of Amsterdam, 1018 WS Amsterdam, The Netherlands; M.J.Noom@uva.nl

**Keywords:** attachment, dog, owner, relationship, scale, questionnaire, styles, secure, insecure, behaviour

## Abstract

**Simple Summary:**

The Strange Situation Procedure is a laboratory test originally designed to assess the quality of a child’s attachment bond to their mother and is widely used in dogs to assess their attachment bond towards the owner. However, the SSP is time consuming and limits the amount and variety of obtainable data. In order to overcome these limitations, we adapted a three-dimensional parent-report scale, named the Attachment Insecurity Screening Inventory (AISI) 6–12, originally developed to assess 6- to 12-year-old children’s attachment insecurity, to dog–owner dyads, and we assessed scale consistency and validity. A first statistical analysis performed on the responses provided by 524 female owners to the online questionnaire revealed five scale dimensions named “physical contact”, “control”, separation anxiety”, “owner as emotional support”, and “owner as a source of positive emotion”. However, a further forced extraction of three components resulted in subscales that mirrored the ones reported for the original AISI in terms of item composition (i.e., ambivalent, avoidant, and disorganized). The three subscales also had satisfactory to good measures of internal reliability. The final scale was named the Dog Attachment Insecurity Screening Inventory (D-AISI). Although promising, it needs to be refined and tested for more validity measures.

**Abstract:**

To date, the Strange Situation Procedure is the only tool available to investigate the quality of the dog’s attachment bond towards the owner. This study aimed to adapt a parent-report scale, named the Attachment Insecurity Screening Inventory (AISI) 6–12, originally designed to assess 6- to 12-year-old children’s attachment insecurity, to dog–owner dyads and assess measures of consistency and validity. The online questionnaire was completed by 524 female dog owners. Principal component analysis (PCA) revealed five components named, respectively, “physical contact”, “control”, “separation anxiety”, “owner as emotional support”, and “owner as a source of positive emotion”. Because of the three-factor structure of the original AISI, a PCA with a pre-fixed set of three factors was also performed. The resulting subscales mirrored the ones found for the original scale (i.e., ambivalent, avoidant, and disorganized), although four items did not fit the model. Internal reliability appeared to be satisfying for the ambivalent and the disorganized subscales, and good for the avoidant subscale. The theoretical background and the results of this study suggest that the three-dimensional model represents a better solution for the interpretation of the Dog Attachment Insecurity Screening Inventory (D-AISI). Although promising, this scale requires refinement and assessment of additional validity measures.

## 1. Introduction

Previous studies have demonstrated that dogs can form an attachment bond with their owners [1]. Taking into account the asymmetry that characterizes the roles of the two parties involved in this relationship, as well as the owner’s perception of their own caregiving role towards their dogs, it was hypothesized that the dog–owner bond may share similar features with that between a child and his caregiver [2]. To date, the scientific focus has shifted from investigating the presence of a bond to assessing its quality or, in other words, whether dogs show different attachment patterns towards their owners, as children do towards their caregivers. The few anthrozoological studies performed so far seem to suggest that the four-style classification scheme used in human infants—categorizing them into either secure, insecure-avoidant, insecure-ambivalent, or disorganized—may be applied to dogs, as well [3,4,5]. Schöberl et al. [3] and Solomon et al. [4] were the first to adapt this qualitative classification to dogs and observe each of the four attachment patterns in a laboratory setting. A more recent study by Riggio et al. [5] provided evidence of a secure and an avoidant-like attachment pattern in dogs by assessing quantitative measures of behaviour during the Strange Situation Procedure (SSP) in subjects that had been classified through a qualitative approach.

So far, dogs’ attachment patterns towards their owners have been investigated with the SSP, which is considered the golden standard for the assessment of children’s attachment styles towards their caregiver. The SSP is a 20 min long semi-structured laboratory procedure designed to progressively increase the level of stress of the tested individual and consequently activate his attachment system towards the caregiver [6]. As for children, the sources of stress for dogs tested in the SSP are represented by the experimental room—in which the whole test takes place—and, most importantly, by two bouts of separations and reunions with the attachment figure [7]. However, as opposed to the case for children, the presence of a stranger, which in this procedure is usually played by a researcher unknown to the tested individual, does not seem to represent an additional source of stress [8,9]. Overall, the SSP is time consuming and requires extensive training for the observer to be able to identify the different attachment patterns [10]. In order to overcome these practical problems, Polderman and Kellaert-Knoll [11] developed the Attachment Insecurity Screening Inventory (AISI), a parent-report questionnaire aimed at assessing human infants’ attachment insecurity towards the parents. The original version was developed for 2- to 5-year-old children and consisted of 20 items investigating specific behaviours of the child and pertaining to three subscales: insecure-avoidant, insecure-ambivalent, and disorganized. Parents indicated the frequency of display of the behaviour described in each item on a response scale from 1 (never) to 6 (always). The questionnaire was shown to have good construct validity, as well as overall good internal reliability. The validity of this new research tool was further supported by the negative correlation with parental sensitivity and the positive correlation with child psychopathology [10]. Not less importantly, it showed a good discriminating power between securely and insecurely attached children assessed through the Attachment Q-Sort (AQS) [10], a validated and largely used measure of attachment security in children [12]. A more recent version of the AISI was proposed for children ranging from 6 to 12 years (AISI 6–12 years) that differed from the previous version in the final number of items—12 out of the original 20 items were retained for an improved factor structure—as well as in the way in which similar aspects of the child–caregiver relationship were investigated according to the distinct interaction dynamics that could be observed between children of these two age groups and their caregivers (e.g., older children are less frequently picked up) [13]. The AISI 6–12 has good reliability for the total scale and satisfactory to good reliability depending on the subscale. No other dimensions of validity were assessed in this case [13].

To date, no similar tool is available for assessing the quality of the dog’s bond towards the owner. For such a purpose, anthrozoology researchers have to rely solely on the use of SSP, which, being a time-consuming procedure, is likely to reduce the variability of the dyads tested, with a bias towards those owners who have a specific interest in the topic or a strong will to commit time and effort to their dogs. Furthermore, the SSP is a laboratory test that does not provide information on dog–owner interactions in a natural environment. This is possibly a major obstacle against a comprehensive understanding of the dog–owner bond [4]. In fact, in human psychology, the final classification scheme of the infant attachment pattern, as well as the SSP were developed after years of ethological observations of child–caregiver interactions in natural settings [6]. This did not occur in the case of dogs, for which the attachment style classification used for human infants was assumed to be suitable based on similarities with the child–mother relationship. Despite the limitations due its caregiver-report nature, a tool such as the AISI may provide a first viable method to investigate dog attachment behaviours towards the owner in daily life. Therefore, the aim of this study was to adapt the initial 20 items of the AISI 6–12 for use in dog–owner dyads and to undertake the first steps to assess the scale’s construct validity on a sample of Italian female owners. It was hypothesized that the questionnaire for the dog–owner dyads had the same factor structure as the questionnaire for the caregiver–child dyads: ambivalent attachment, avoidant attachment, and disorganized attachment.

## 2. Materials and Methods

This study received a favourable recommendation by the Committee on Bioethics of the University of Pisa, Italy (review no. 6/2021).

### 2.1. Owners’ and Dogs’ Demographics

As a first step in the validation of the dog-adapted version of the AISI, only the responses from female owners were considered for analysis. A total of 524 female owners aged between 18 and 72 years old (mean = 39.0, SD = 11.1) completed the questionnaire. More than half of them (50%) had either a graduate or a post-graduate degree. Concerning their occupation, 5.2% of them were not occupied at the moment of their participation in the study, 1.9% were retired, 9.2% were students, and 24.5% had a profession that involved animals (e.g., animal health professionals, animal trainers, breeders). For 46.9% of them, the first experience with dog ownership occurred in their childhood, for 24.2% it was in their adolescence and for 28.8% in their adulthood. The number of dogs owned before the current one ranged between 0 and 29 (median = 2).

As for the dogs’ demographics, 54.6% of them were females and 61.5% were neutered. Their age ranged from 0.5 to 18 years (mean = 6.4, SD = 4.1). The most represented dog size was medium (44.5%), followed by large (26.5%), small (22.1%), mini (5.3%), and giant (1.5%). Almost half of them were mix-breed (49.2%), while the most represented breeds were Cavalier King Charles Spaniel (5.6%), Border Collie (3.6%) Labrador Retriever (2.7%), Jack Russell (2.3%), American Staffordshire Terrier (2.3%), Australian Shepherd (2.1%), and Golden Retriever (2.1%). Finally, 91.4% of the dogs were reported to be physically healthy at the time of the study.

### 2.2. Measurements

The whole questionnaire consisted of four different sections. The first section aimed at collecting demographic data about the respondents (age, gender, profession, education level, dog ownership experience, etc.) and the dogs (breed, size, sex, age, neuter status, physical and behavioural problems, etc.). The second section consisted of a dog-adapted version of the initial 20 items from the AISI 6–12 for children. The third section was composed of items from the Parenting Styles and Dimensions Questionnaire (PSDQ) for dogs [14]. Finally, the fourth section focused on collecting information on the presence of behavioural disorders (e.g., phobias, anxiety, aggressiveness, compulsive behaviours) and/or problematic behaviours (e.g., disobedience, house soiling) in the dogs. Responses from the third and the fourth section were not analysed in the current study. All questions were written in Italian.

### 2.3. Development of the Dog Attachment Insecurity Screening Inventory (D-AISI)

All the initial 20 items from the AISI 6–12 [13] were first independently translated into Italian by two Italian native speakers who were also proficient in English and had deep knowledge of the attachment theory. The two translated versions were then compared, and incongruities were discussed to reach consensus. In order to avoid possible misinterpretations, uncertainties on the meaning of some items were discussed with one of the authors of the original AISI 6–12. Afterwards, the questionnaire was back translated to confirm the accuracy of the Italian version. The latter was then adapted to dogs through a multistep process. Firstly, the word “child” was replaced with the word “dog”. Secondly, some words that had been chosen to describe children’s behaviour in the original AISI but did not sound appropriate to depict canine behaviour, were changed without altering the general meaning of the item. At this point, a draft of the questionnaire was administered to a pilot sample of 10 dog owners who provided feedback on the lack of clarity of some items/responses. Finally, according to this feedback, some actual examples of the dog behaviour were added at the end of the respective items. Furthermore, the original 1 to 6 response scale (1 = never; 2 = sometimes; 3 = regularly; 4 = often; 5 = very often; 6 = always) used in the AISI 6–12 for children was replaced by a 1 to 5 scale (1 = never; 2 = rarely; 3 = sometimes; 4 = often; 5 = always), in order to provide a neutral response option and a more straightforward labelling of the scale points. The final scale was named the Dog Attachment Insecurity Screening Inventory (D-AISI). A list of the items is reported in Table 1.

### 2.4. Procedure

The questionnaire was developed using Jotform^®^ (San Francisco, CA, USA) and shared on social media platforms, such as Facebook^®^ (http//www.facebook.com, accessed on 24 October 2021) and Instagram^®^ (http//www.instagram.com, accessed on 24 October 2021), as well as directly e-mailed to personal contacts of the authors. All the responses were collected in the month of April 2021. Respondents were required to (1) be dog owners, (2) be at least 18 years old, and (3) have agreed to the informed consent. If they owned more than one dog, they were asked to answer for the dog they had been living with for the longest time.

### 2.5. Statistical Analysis

The statistical analyses were carried out using the Statistical Package for the Social Sciences (SPSS, IBM, New York, NY, USA) 17.0. First, the responses collected from the AISI were checked for low standard deviation (SD < 0.5) and high skewness and kurtosis (above 6), which led to the exclusion of one item. Then, a principal component analysis (PCA) was applied to the remaining 19 items. Five factors were extracted with varimax rotation. However, considering the original AISI three-dimensional model and the visualization of the scree plot, a second PCA was performed with a pre-set extraction of three components. In order to assess the internal reliability, Cronbach’s α values were calculated for each subscale of both the five-dimensional and the three-dimensional model, as well as for the entire final scale, as performed for the original AISI [10].

## 3. Results

### 3.1. The Five-Dimensional Model of the D-AISI

A first check of the 20 items for skewness, kurtosis, and low variance resulted in the exclusion of item 18, “Does your dog get angry with you out of proportion and/or without apparent reasons?” (see Table S1 for a summary of descriptive statistics of the items). The remaining 19 items were analysed using a PCA with varimax rotation. The Kaiser–Meyer–Olkin measure of sampling adequacy was 0.80, above the commonly recommended value of 0.60, and Bartlett’s test of sphericity was significant (χ^2^ (171) = 2151.05, *p* < 0.001). In order to be retained in the scale, the items’ loadings had to be ≥0.4 on the primary component and ≤0.35 on the others. Five principal components (PC1, PC2, PC3, PC4, and PC5) were identified that explained 53.933% of the variance (PC1: 14.73%, PC2: 11.82%, PC3: 10.03%, PC4: 9.58%, and PC5: 7.79%). The factor loading matrix for this final solution is reported in Table 1. PC1 was composed of items relating to the dog’s attitude and responses to physical contact with the owner. PC2 comprised those items relating to the dog’s tendency to control the daily dynamics of interaction with the owner. PC3 was composed of items regarding the dog’s separation anxiety. PC4 included those items relating to the dog’s tendency to use the owner as a source of emotional support. Although item 8 had a secondary loading >0.35 in PC1, it was not excluded from the final scale because of its quite high primary loading on PC4. Finally, PC5 comprised items describing the owner as a source of positive emotion for the dog.

The Cronbach’s α values were calculated for each subscale, showing good internal reliability for the “physical contact” subscale (α = 0.765), moderate for the “control” subscale (α = 0.667), poor for the “separation anxiety” (α = 0.599), and “owner as emotional support” (α = 0.589) subscales and unsatisfactory reliability for the “owner as a source of positive emotion” subscale (α = 0.488).

### 3.2. The Three-Dimensional Model of the D-AISI

Taking into account the three-factor model found in the original scale [13] and the scree plot curve, the 19 items from the questionnaire—item 18 was again excluded—were also analysed using a PCA with varimax rotation forced to extract three components. The Kaiser–Meyer–Olkin measure of sampling adequacy was 0.80, and Bartlett’s test of sphericity was significant (χ^2^ (171) = 2151.5, *p* < 0.001). In order to be retained in the scale, the items’ loadings had to be ≥0.4 on the primary component and ≤0.35 on the others. The three principal components explained 42.090% of the variance (PC1: 17.22%, PC2: 12.98%, PC3: 11.89%) and reflected those in the original study, except for three items (2, 9, and 15) that fell into different subscales (Table 2). Specifically, PC1, which was named “avoidant”, was composed of all the items belonging to the original avoidant subscale, except for item 9, which fell into the ambivalent subscale, and with the addition of item 15, which originally pertained to the ambivalent subscale. Since item 11 scored just below the cut-off point of 0.4 it was not excluded from the final scale. PC2, which was named “ambivalent” was composed of all the items belonging to the original ambivalent subscale except for item 2, which fell into the disorganized subscale, and items 9 and 15. Finally, PC3, which was named “disorganized”, was composed of all the items belonging to the original disorganized subscale with the addition of item 2 (Table 3).

Cronbach’s α values were calculated as a measure of internal reliability of each subscale, which appeared to be good for the avoidant subscale (α = 0.779) and moderate for both the ambivalent (α = 0.660) and the disorganized (α = 0.667) subscales. In accordance with the procedure reported by Wissink et al. [10], internal reliability was assessed for the total attachment insecurity scale. In this case, Cronbach’s α was slightly lower (α = 0.605).

## 4. Discussion

The aim of this pilot study was to assess whether a dog-adapted version of the original 20 items of the AISI 6–12, developed to assess parent’s perception of the child’s attachment behaviour, was also suitable to investigate the quality of the dog’s bond towards the owner.

A first attempt to identify the internal structure of the D-AISI was made by allowing the PCA to freely generate a set of dimensions. The result was a scale composed of five different dimensions or subscales that described different features of the dog’s attachment behaviour towards the owner. Based on the original AISI’s three-dimension model, we then decided to force the PCA to extract a total of three components. The outcome was a set of subscales that were highly consistent with the ones reported for the original scale used for children, except for four items that fell into different subscales. Overall, the three-dimensional model seems to represent a better solution for the interpretation of the D-AISI. In order to provide a clearer explanation of the process the led to the development of the final scale, in this section, we discuss the conceptual links that tie each subscale of the five-dimensional model to the ones found in the preferred three-dimensional D-AISI. Furthermore, we highlight differences and similarities between the three-dimensional D-AISI and the AISI 6–12 [13], and we provide possible explanations for the items that did not fit the original model.

As previously mentioned, the first subscale of the five-dimensional D-AISI was named “physical contact” as it related to the dog’s attitude towards interactions with the owner involving physical contact. All the items in this subscale can also be found in the avoidant subscale of the final three-dimensional D-AISI. This finding is conceptually coherent with the child attachment style classification in which the term “avoidant” is used to label those children who tend to avoid proximity and interaction with the attachment figure [6,15]. Similarly, in the dog attachment classification used by Schöberl et al. [3] and Solomon et al. [4], avoidant dogs are described as individuals who show little tendency to approach, seek contact, and interact with the caregiver during the SSP. Furthermore, the presence of an avoidant-like attachment pattern in dogs has been demonstrated by Riggio et al. [5], who found that, contrary to securely attached dogs, subjects classified as avoidant do not increase their proximity or their contact seeking behaviour towards the owner across SSP episodes. Therefore, considering that the “physical contact” dimension is particularly relevant for the identification of the avoidant attachment pattern in both children and dogs, it is not surprising that all the items of this subscale fall into the avoidant subscale of the three-dimensional AISI for dogs. However, item 15 “Does your dog want to be left alone and simultaneously seeks contact with you (e.g., asks to be petted but then leaves or growls)?” fell into the avoidant subscale for D-AISI and the ambivalent subscale for AISI 6–12. One possible explanation is that, despite the similarities with the avoidant subscale of the AISI 6–12, what we named the avoidant subscale in the D-AISI does not actually measure avoidance as a whole, but rather a specific dimension of the avoidant attachment pattern, which is the dog’s (lack of) manifestation of pleasure in response to interactions with the caregiver. On the other hand, it is also possible that ambivalent dogs do not tend to show overtly resistant behaviour towards the owner, as suggested by Solomon et al. [4]. This hypothesis will be discussed later in the manuscript.

The second subscale of the five-dimensional D-AISI was named “control” as it related to the dog’s tendency to impose himself on the outcome of routine activities and maintain control over the dynamics of interaction with the owner. In this case, all the items that compose this subscale can be found in the disorganized subscale of the three-dimensional D-AISI. Again, this finding is coherent with the attachment style classification used for children. Previous studies [16,17] report that approximately two-thirds of pre-school children with disorganized attachment develop role reversal in later stages of growth. Role reversal is a condition in which the attached figure leads the dynamics of interaction with the attachment figure [18]. In the child–mother relationship, this occurs when the attachment figures are unable to fulfil their caregiving role and to provide protection, support and guidance to the child [19].

It would be interesting for future studies to assess whether a dog’s tendency to control may be associated with the owner’s helplessness and incapability to assume the role of caregiver. This may have enormous implications on the rehabilitative approach that canine behavioural professionals use to intervene in those problematic behaviours that entail some degree of conflict with the owner. Nowadays, a dog’s controlling behaviour towards the owner is approached with either a psychopathological perspective—controlling behaviour as symptom of anxiety in response to an unpredictable environment—[20] or, even more commonly, with an eco-ethological perspective—controlling behaviour in terms of agonistic interactions aimed at establishing a dominant–submissive relationship [21,22]. While each case should be evaluated individually, an attachment-based approach may offer new interpretative models to this problematic behaviour [23], as well as additional strategies for intervention.

Overall, the item composition of the disorganized subscale of the three-dimensional D-AISI is very similar to the one reported for children in the AISI 6–12. Only two items, namely item 2 “Is your dog excessively docile and compliant?” and item 18 “Does your dog get angry with you out of proportion and/or without apparent reasons?” did not fit the model. The latter was excluded before statistical analysis because of its high skewness to the right, meaning that the distribution of the responses was not normal and that the majority of them gathered on the lower end of the scoring scale. Differently, item 2 was not removed but fell into the disorganized subscale instead of the ambivalent subscale, as observed in children. Possibly, this discrepancy may be explained by the choice of words used in the translated version of the item, where the word “obedient”—“ubbidiente” in Italian–was purposefully replaced by the word “remissivo”, which describes a compliant and submissive individual [24]. This change was implemented because high levels of obedience are often regarded by owners as the most desirable feature of their dog’s behaviour. Hence, we believed it would have been difficult for them to picture their dog as “excessively obedient”. Regardless of the reason, the presence of an item about the dog’s compliance and docility in the “disorganized subscale” of the three-dimensional D-AISI, which deals entirely with the dog’s tendency to control the dynamics of interaction with the owner, seems quite logical.

The third subscale was labelled “separation anxiety” as it seemed to describe the dog’s behaviour in response to actual or possible separation from the owner. All the items in this subscale fall in the ambivalent subscale of the three-dimensional D-AISI and are also found in the same subscale of the AISI for children. These findings are in line with current scientific literature on child insecure-ambivalent attachment, which is often reported to be strongly associated with anxiety disorders [25,26,27,28]. Mothers of ambivalent attached children tend to display an inconsistent and irregular caregiving behaviour that generates a feeling of unpredictability on their availability in times of need [29]. This is the reason why ambivalent children are described as chronically vigilant, scarcely autonomous, poorly interested in environmental exploration, and extremely upset by separation from the attachment figure [30]. Further support for the theoretical link between the “separation anxiety” and “ambivalent” subscales of the two models of the AISI for dogs comes from a series of studies carried out by Konok et al. [31,32,33]. In fact, their findings suggest that dogs with separation anxiety show an ambivalent attachment behaviour towards their owners [32], who in turn tend to be less sensitive and responsive compared to those of securely attached dogs [31,33].

The fourth subscale was named “owner as emotional support”. Amongst all the subscales, this was the most challenging to interpret. In fact, at first glance, item 10 “Does your dog seem very concerned for you when you are upset or unwell?” may not seem to relate to the role of the owner as emotional support. However, based on previous literature on infant attachment, caregiver’s conditions of emotional upset or physical illness, even if transitory, may negatively affect his/her availability to the child’s needs, as well as the quality of the response to the child’s support seeking behaviours [34]. In these circumstances, a child’s extreme concern is likely to reflect the fear of losing a source of emotional support and, according to previous literature, may be associated with attachment insecurity [34]. Although, on the one hand, the “emotional support” dimension may offer the most comprehensive interpretation of the construct behind this subscale, on the other, the “insecurity” dimension seems to provide a logical explanation for the distribution of these items into different components in the three-dimensional D-AISI. While items 9, 10, and 20, which describe different facets of the dog’s insecurity and lack of self-confidence when dealing with external challenges, fall into the ambivalent component, item 8, which instead focuses on the dog’s response to proximity to and contact with the owner (this is probably the reason why it also loads relatively high on the “physical contact” subscale), falls into the avoidant component. The distribution of these items in the three-dimensional D-AISI reflects that of the AISI for children, except for item 9 “Does your dog ask for help when he/she has a problem (e.g., if scared of something, cannot reach something he/she is interested in, etc.)?”. Although the behaviour described in this item may theoretically relate to both the avoidant and the ambivalent dimensions of attachment—indeed, we would expect opposite responses between avoidant and ambivalent individuals—ambivalent children are reported to show higher levels of attention, as well as active social referencing behaviour towards the caregiver, in times of distress [35,36]. These behavioural features of ambivalent children clarify the presence of this item in the ambivalent subscale of the D-AISI.

Finally, the fifth subscale was labelled “owner as a source of positive emotion” as it related to the dog’s tendency to manifest joy in the presence of the owner. It makes sense that both the items that compose this dimension fall into the avoidant subscale in the three-dimensional D-AISI. One may argue that both ambivalent and avoidant children may display behaviours that are not indicative of a positive emotional state in the presence of the caregiver. However, the behavioural expression of the former, which is characterized by an evident and intense need for proximity and contact, and simultaneous active attempts of rejection, may be more difficult for parents to interpret [13]. On the contrary, the avoidant children’s apparent emotional and behavioural indifference to the caregiver’s presence may be more easily interpreted as the lack of a joyful response. This may be particularly true when the behaviour of a different species has to be interpreted. In fact, dog ambivalent patterns seem to be the most problematic to identify even for anthrozoology researchers [4,5]. The percentage of ambivalent dogs reported in previous studies was extremely variable, ranging from 4% [5] to almost 44% [37]. As suggested by Solomon et al. [4], one major problem with the identification of the ambivalent attachment pattern in dogs may be the absence of clear signs of angry resistance, which is the core feature of this attachment style in children. As suggested by previous authors, this may be the outcome of a millenary process of dog domestication that selected against angry and conflicting behaviour towards humans [4], or the reflection of a different behavioural expression of ambivalence in the canine species [38], or still, the consequence of testing adult individuals [38,39], in who may display ambivalent/resistant behaviours in a more subtle and passive manner than young children might [13,36]. However, regardless of the cause, the lack of an overt ambivalent/resistant behaviour in dogs may explain why, in the three-dimensional model of the D-AISI, item 15, which is the only item that specifically addresses resistant behaviour, does not share the same construct with other items that describe the ambivalent attachment pattern.

Although not definitive, the results of this study represent a first step towards the development of a scale that may help anthrozoology researchers avoid those limitations that stem from the sole use of a laboratory test, such as the SSP, for the investigation of dog attachment behaviour. Firstly, by reducing the commitment of dog owners to the provision of data, a tool such as the current questionnaire may prompt the participation of individuals who may refuse to be involved in more time-consuming procedures, therefore increasing the variability and the total amount of obtainable data. Secondly, by focusing on dog attachment behaviour from daily interactions in natural contexts, it provides information that could not be obtained with the use of a laboratory test. In fact, observations of child–caregiver interactions in a natural environment provided a solid theoretical background for the development of the infant attachment style classification, as well as for the validation of the SSP as an instrument to identify different styles. This type of information on dog–owner interactions is not yet available and would require incredibly long times to be collected through ethological observations.

The present study has some limitations. First of all, being a questionnaire-based study, the information obtained reflects the respondent’s subjective interpretation of the dog’s attachment behaviour and emotional state. Previous findings seem to suggest that dog owners may not always be able to recognize dogs’ emotional expressions. For instance, Tami et al. [40] found that owners tend to confuse aggressive with play behaviour—and vice versa—and also tend to misinterpret—or mislabel—submissive behaviour as friendliness. Demirbas et al. [41] suggest that owners may struggle in recognizing dogs’ emotional expressions of fear and anxiety during an interaction with a third party. Moreover, Mariti et al. [42] found that dog owners tend not to recognize subtle indicators of stress (e.g., turning the head, avoid looking, nose-licking, yawning), possibly misinterpreting the dog’s emotional state during interspecific interactions. This is an aspect that should be taken into account if an owner-report scale aimed at identifying dog attachment insecurity will be used for either research or clinical purposes in the future. Certainly, future studies will have to assess other measures of this scale’s validity—such as concurrent and convergent validity—that have not been tested in this study and that may support or confute the suitability of this scale in dog attachment research.

A second limitation of this study is the absence of a sample of male owners. Spruit et al. [13] demonstrated that the internal structure of the original AISI 6–12 is not affected by the gender of the respondents and that mothers and fathers interpret the items in the same way [13]. However, it should not be assumed that this would also be the case for female and male owners. Findings from several previous studies suggest that women and men have different perceptions of animal emotions and behaviours [41,43,44,45,46,47], which may alter the pattern of responses provided and, consequently, the structure of the scale. Therefore, future studies should assess whether the statistical model of the D-AISI currently observed would be suitable to explain the pattern of responses provided by male owners, as well.

A third limitation is that factors related to the dogs, such as breed, age, sex, and past experiences may affect the way they behave towards the owner [8,48,49,50,51,52]. A study including a very large and heterogeneous sample is needed to explore these variables in more depth.

Finally, this study entailed both a process of adaptation of the scale from the human species to the canine species and a process of translation from English to Italian. Although we took extreme care in retaining the original meaning of the items, misinterpretations due to language differences and due to the conceptual adaptation of the items to a different species may still occur. This should be taken into account for future refinements of this scale.

## 5. Conclusions

This pilot study provides information on the internal structure and consistency of an owner-report scale to assess dog attachment insecurity. The internal structure of the D-AISI appears to be similar to that reported in the original study on children. However, a few items did not fit the original model. Reliability coefficients for the subscales of the five-dimensional model were not always acceptable. On the contrary, they were deemed satisfying for each subscale of three-dimensional D-AISI, as well as for the entire scale. Overall, the three-dimensional D-AISI seems to provide a more valid and reliable model than the five-dimensional solution to investigate dog attachment styles. Indeed, this scale needs to be refined, and other measures of validity need to be investigated, before it can be used to assess the quality of a dog’s attachment to their owner. Nonetheless, the development of such a tool may have several positive implications for dog-attachment research, such as obtaining information on attachment behaviours in a natural context that cannot be investigated by means of the SSP, increasing the variability and the overall amount of data acquired, by including owners who may not be willing to be involved in time-consuming laboratory procedures, and generating data that are easy and quick for researchers to collect and interpret.

## Figures and Tables

**Table 1:** Rotated component matrix for the five-component solution.

Item	Principal Component				
	PC1	PC2	PC3	PC4	PC5
14. Does your dog enjoy physical contact with you? (R)	0.762 *	0.004	−0.113	0.090	0.201
3. Does your dog respond positively and remain relaxed when you touch him/her? (R)	0.737 *	0.132	−0.011	0.054	0.088
5. Does your dog like to be cuddled by you? (R)	0.702 *	0.126	−0.264	−0.017	0.182
15. Does your dog want to be left alone and simultaneously seeks contact with you (e.g., asks to be petted but then leaves or growls)	0.674 *	0.151	0.180	0.086	−0.098
17. Does your dog reach out spontaneously to cuddle with you? (R)	0.527 *	−0.091	−0.193	0.182	0.292
1. Does your dog try to force you to do what he/she wants?	−0.036	0.735 *	0.149	0.025	0.117
7. Does your dog try to impose himself over you if things do not turn out the way he/she expects? (e.g., expects a treat, expects to go to the park, but you go another direction)	0.002	0.702 *	0.099	−0.064	0.335
12. Is your dog extremely determined to decide everything for himself/herself?	0.067	0.649 *	−0.051	0.090	−0.205
4. When you play with your dog, does it seem like he/she wants to be in control of the dynamics of the game?	0.168	0.613 *	0.131	−0.129	0.112
2. Is your dog excessively docile and compliant?	−0.218	−0.508 *	0.257	−0.132	0.134
16. Does your dog keep an eye on you while you do things in and around the house?	−0.064	−0.002	0.765 *	−0.091	0.024
6. Does your dog always stay close to you?	−0.229	−0.072	0.653 *	−0.245	−0.257
13. Does separation from you cause extremely strong emotional reactions in your dog?	0.035	0.208	0.647 *	−0.020	−0.046
9. Does your dog ask for help when he/she has a problem (e.g., if scared of something, if he/she cannot reach something is interested in) (R)	0.101	−0.084	−0.020	0.814 *	0.082
20. In your opinion, does your dog need you to reassure him/her that he/she is doing something right? (e.g., before approaching a dog or a person)	0.002	−0.118	0.266	−0.591 *	0.077
8. Does your dog let you comfort him/her when he/she is in pain, frightened, or upset? (R)	0.422	0.059	0.125	0.564 *	0.085
10. Does your dog seem very concerned for you when you are upset or unwell?	−0.031	0.075	0.297	−0.514 *	−0.355
11. Does your dog positively interact with you after you have been away for a short period of time? (R)	0.172	0.088	−0.011	−0.001	0.754 *
19. Is your dog happy and playful in your presence? (R)	0.304	0.085	−0.173	0.241	0.567 *

^1^ The highest loadings for each item are marked with a *. ^2^ (R) = reversed item.

**Table 2:** Rotated component matrix for the three-component solution.

Item	Principal Component		
	PC 1	PC 2	PC 3
14. Does your dog enjoy physical contact with you? (R)	0.783 *	−0.104	−0.005
3. Does your dog respond positively and remain relaxed when you touch him/her? (R)	0.725 *	0.016	0.117
5. Does your dog like to be cuddled by you? (R)	0.698 *	−0.163	0.107
15. Does your dog want to be left alone and simultaneously seeks contact with you (e.g., asks to be petted but then leaves or growls)	0.622 *	0.178	0.131
17. Does your dog reach out spontaneously to cuddle with you? (R)	0.599 *	−0.262	−0.085
8. Does your dog let you comfort him/her when he/she is in pain, frightened, or upset? (R)	0.506 *	−0.201	0.077
19. Is your dog happy and playful in your presence? (R)	0.487 *	−0.351	0.123
11. Does your dog positively interact with you after you have been away for a short period of time? (R)	0.399 *	−0.131	0.141
6. Does your dog always stay close to you?	−0.291	0.681 *	−0.070
16. Does your dog keep an eye on you while you do things in and around the house?	−0.020	0.645 *	0.028
10. Does your dog seem very concerned for you when you are upset or unwell?	−0.194	0.596 *	0.040
20. In your opinion, does your dog need you to reassure him/her that he/she is doing something right? (e.g., before approaching a dog or a person)	−0.038	0.536 *	−0.123
13. Does separation from you cause extremely strong emotional reactions in your dog?	0.053	0.535 *	0.226
9. Does your dog ask for help when he/she has a problem (e.g., if scared of something, if he/she cannot reach something he/she is interested in) (R)	0.230	−0.493 *	−0.051
1. Does your dog try to force you to do what he/she wants?	0.013	0.085	0.748 *
7. Does your dog try to impose himself over you if things do not turn out the way he/she expects? (e.g., expects a treat, expects to go to the park, but you go another direction)	0.104	0.059	0.726 *
12. Is your dog extremely determined to decide everything for himself/herself?	0.005	−0.041	0.629 *
4. When you play with your dog, does it seem like he/she wants to be in control of the dynamics of the game?	0.182	0.180	0.614 *
2. Is your dog excessively docile and compliant?	−0.163	0.228	−0.483 *

^1^ The highest loadings for each item are marked with a *. ^2^ (R) = reversed item.

**Table 3:** Item composition for the subscales of the Attachment Insecurity Screening Inventory (AISI) and the Dog Attachment Insecurity Screening Inventory (D-AISI).

Subscale	Items	
	AISI	D-AISI
Avoidant	3, 5, 8, 9, 11, 14, 17, 19	3, 5, 8, 11, 14, 15 *, 17, 19
Ambivalent	2, 6, 10, 13, 15, 16, 20	6, 9 *, 10, 13, 16, 20
Disorganized	1, 4, 7, 12, 18	1, 2 *, 4, 7, 12

^1^ Items of the D-AISI that did not fall into the original subscale are marked with a *. ^2^ Item 18 was removed from the D-AISI.

## Data Availability

The data presented in this study are available on request from the corresponding author.

## Supplementary Materials

The following are available online at https://www.mdpi.com/article/10.3390/ani11123381/s1, Table S1: Descriptive statistics of the items.
