# Supplementary material for: Development of the Dog Attachment Insecurity Screening Inventory (D-AISI): A Pilot Study on a Sample of Female Owners
Source: Animals (Basel). 2021 Nov 26;11(12):3381. doi: 10.3390/ani11123381 (PMC8698140; doi:10.3390/ani11123381)
Supplement: Supplementary file 1 [file animals-11-03381-s001.zip › animals-1469819-supplementary.pdf]

Table S1: Descriptive statistics of the items

Item	Min.	Max.	Mean	Standard Deviation	Skewness	Kurtosis
1	1	5	2.52	0.901	0.075	-0.285
2	1	5	3.43	1.094	-0.315	-0.500
3	1	4	1.42	0.660	1.461	1.475
4	1	5	2.61	1.081	0.078	-0.660
5	1	4	1.44	0.707	1.501	1.528
6	1	5	4.14	0.813	-0.535	-0.460
7	1	5	2.26	0.998	0.331	-0.705
8	1	5	1.70	0.904	1.199	0.838
9	1	5	1.91	0.936	0.892	0.339
10	1	5	3.58	1.056	-0.408	-0.393
11	1	3	1.21	0.470	2.246	4.372
12	1	5	2.56	1.002	0.312	-0.279
13	1	5	2.57	1.160	0.376	-0.746
14	1	4	1.48	0.684	1.166	0.338
15	1	5	1.64	1.018	1.490	1.214
16	1	5	3.68	1.144	-0.576	-0.508
17	1	5	1.91	0.788	0.685	0.326
18	1	4	1.16	0.494	3.567	13.979
19	2	5	4.41	0.658	-0.715	-0.367
20	1	5	2.75	1.054	0.242	-0.515
